# Anti-neoplastic Potential of Flavonoids and Polysaccharide Phytochemicals in Glioblastoma

**DOI:** 10.3390/molecules25214895

**Published:** 2020-10-23

**Authors:** Ayesha Atiq, Ishwar Parhar

**Affiliations:** 1 Brain Research Institute Monash Sunway (BRIMS), Jeffery Cheah School of Medicine, Monash University Malaysia, 47500 Bandar Sunway, Selangor, Malaysia; Ayesha.atiq@monash.edu; 2 Brain Research Institute Monash Sunway (BRIMS), Jeffrey Cheah School of Medicine and Health Sciences, Monash University Malaysia, 47500 Bandar Sunway, Selangor, Malaysia

**Keywords:** glioblastoma, flavonoids, polysaccharides, phytochemicals, anti-neoplastic

## Abstract

Clinically, gliomas are classified into four grades, with grade IV glioblastoma multiforme being the most malignant and deadly, which accounts for 50% of all gliomas. Characteristically, glioblastoma involves the aggressive proliferation of cells and invasion of normal brain tissue, outcomes as poor patient prognosis. With the current standard therapy of glioblastoma; surgical resection and radiotherapy followed by adjuvant chemotherapy with temozolomide, it remains fatal, because of the development of drug resistance, tumor recurrence, and metastasis. Therefore, the need for the effective therapeutic option for glioblastoma remains elusive. Previous studies have demonstrated the chemopreventive role of naturally occurring pharmacological agents through preventing or reversing the initiation phase of carcinogenesis or arresting the cancer progression phase. In this review, we discuss the role of natural phytochemicals in the amelioration of glioblastoma, with the aim to improve therapeutic outcomes, and minimize the adverse side effects to improve patient’s prognosis and enhancing their quality of life.

## 1. Introduction

Glioblastoma multiforme (GBM) is a malignant brain tumor that is highly invasive in nature with a high proliferation rate and extremely vascularized tumor blood supply, which makes it highly aggressive among other cancers [1,2]. Despite advancements in molecular biology and genetics of cancer, GBM still poses a therapeutic challenge, and improvements in prognosis remain poor with a survival of 10–12 months on average [3]. The World Health Organization has classified gliomas into four grades ranging from low-grade astrocytomas and oligodendrogliomas to high-grade astrocytomas or glioblastoma (GBM) based on genetics and histopathological parameters [4]. GBM is further categorized into primary GBM that originates de-novo from glial cells, or secondary GBM that originates from pre-existing lower-grade astrocytoma. The primary GBM is characterized by overexpression of the epidermal growth factor receptor gene and phosphatase and tensin homolog mutation; however, the hallmark feature of secondary GBM is the loss of the p53 gene and overexpression of platelet-derived growth factor [5,6].

The current treatment modalities include surgical resection of the tumor, followed by chemotherapy and radiotherapy [7,8]. Even with the advancement of surgical techniques, such as fluorescent guided resection and neuroendoscopic approaches, the complete surgical resection of gliomas is challenging, as the tumor cells located at tumor edges in the perivascular niches are mostly left behind [9]. Owing to stem-cell-like properties of most of these cells, such as partial pluripotency, lack of differentiation, and self-renewal potential, there is a likeliness of new tumor growth [10]. Moreover, adjuvant chemotherapy with temozolomide is unable to improve the prognosis for more than 2.5 months as it is not specifically directed toward these stem-like tumor cells. Due to the abrupt invasion and infiltration of adjacent healthy brain tissues, metastasis to distant brain regions is paramount [11]. However, due to the blood-brain barrier (BBB), their dissemination outside the brain is not possible, but their proliferation within the brain itself results in detrimental consequences, including vasogenic and cytotoxic brain edema that disables vital brain centers [12]. 

Temozolomide is a novel oral alkylating agent used for the clinical treatment of GBM. It methylates the O6-position of guanine in double-stranded DNA, causing mismatches with thymine, consequently blocking DNA replication, and triggering cell death [3,13]. O^6^ methyl guanine DNA methyltransferase (MGMT) removes the O6-methylguanine adducts induced by temozolomide and other alkylating agents, thereby repairing the DNA. This DNA repairing causes MGMT protein depletion, which is replenished by the cell. Therefore, higher levels of MGMT are alleged to lead to temozolomide resistance [14]. Furthermore, temozolomide is associated with severe adverse effects on the immune system, causing treatment delay and death [15]. 

Taken together, treatment failures are attributed to amalgamations of cellular heterogeneity, which includes the highly invasive nature of GBM cells, stem cell-like properties, aberrant vasculature, remarkable immune suppression, and developed chemoresistance. Current treatment modalities do not address all these factors satisfactorily [9,16]. Hence, there is an imperious need for the development of novel, multimodal, and effective therapy for GBM. Moreover, multiple genetic alterations in GBM suggest that combined therapy of two or more therapeutic agents are likely to offer more effective management of the disease compared to monotherapy [17]. Besides, the simultaneous targeting of multiple aberrant genetic pathways by a single drug entity is more beneficial [18]. Scientific literature reports that various compounds of the edible plants collectively termed phytochemicals are capable of targeting multiple genetic pathways simultaneously, and prove helpful in the eradication of cancer as a single drug entity [19]. Among the phytochemicals, flavonoids and polysaccharides have been broadly evaluated for their numerous biological functions, such as anti-oxidant, anti-inflammatory, and anti-cancer activities [20,21,22]. Additionally, polysaccharides have gained much attention in immuno-pharmacology research after the discovery of their immunomodulatory and anti-tumor effects in 1950s [23]. 

Chemotherapeutics cause DNA damage, which can cause mutations in normal cells converting them to cancerous cells, thereby increasing the risk of secondary malignancies. Because chemotherapy does not spare normal cells from their devastating action, it has a severe toxic effect on the patients [24]. On the other hand, natural compounds, such as flavonoids and polysaccharides, are generally safer and do not adversely affect the patient, compared to conventional chemotherapy [25,26]. 

In this review, we highlight the advancement in recent research and available data on molecular mechanisms underlying chemopreventive effects of flavonoids and polysaccharides in GBM. We also discuss possible ways to improve bioavailability and drug delivery of phytochemicals to cancer cells. 

### 1.1. Putative Targets in Glioblastoma

#### 1.1.1. Genetic Alterations

Analysis of genetic alterations in GBM has identified three commonly deregulating genetic pathways in glioblastoma; the p53 pathway is the most commonly mutated pathway in tumorigenesis that plays a vital role in the progression of secondary GBMs. P53 (tumor suppressor) responds to DNA damage by inhibiting cell cycle progression and inducing apoptosis [27,28]. In addition, it also regulates the transcription of many genes involved in the development and progression of tumors [27]. The overexpression of mouse double minute 2 homolog (MDM2) inactivates an alternative p53 regulated growth pathway in all malignant GBMs without p53 mutations. Furthermore, the inactivation of the alternative reading frame (ARF), an upstream regulator of the p53 pathway, is commonly found in all gliomas [29].

Mutations in tumor suppressor retinoblastoma (pRB), vital for cell cycle inhibition, are detected in 20% of high-grade gliomas. Considering that the pRB signaling is inhibited by kinase activities of the CDK4/CDK6 and Cyclin D complex, the inactivation of CDK4/6 could be a novel anti-glioblastoma treatment option for GBM patients with aberrant expression of pRB [30]. 

Alterations in the phosphatidylinositol 3 kinase (PI3K) pathway are described in almost 70% s of GBMs, either due to loss of PTEN (inactivates PI3K/AKT/PKB pathway) or amplification of estrogen growth factor receptor, platelet-derived growth factor receptor alpha, or vascular endothelial growth factor receptor [29,31]. Moreover, a defective RAS/MAPK pathway with overexpression of RAS has been identified in multiple cancers, including GBM [27,32].

#### 1.1.2. Autophagy

Autophagy is a catabolic process that is vital to maintain cellular homeostasis by lysosomal degradation of damaged or excess organelles [33]. On the contrary, when cells undergo stress conditions, such as hypoxia and nutrient depletion autophagy, is an effort to maintain their survival. In GBM, the densely packed tumor cells undergo oxygen and nutrient depletion; herein, autophagy has been found to be up-regulated as a protective mechanism to avoid necrotic cell death. In GBM, targeting of autophagy has shown promising results [34]. However, whether inhibition of protective autophagy or over activation of autophagy is more beneficial, still needs to be elucidated. In solid tumors treatment, both methods have been proven as effective. Inhibition of protective autophagy reduces the previously activated defense mechanisms of the cell. Whereas, the induction or over activation of autophagy induces programmed cell death or autophagic cell death through the over activation of lysosomal degradation [35]. A previous report demonstrates enhanced cytotoxicity of temozolomide to glioma cells in combination with thalidomide, a drug that acts through PI3K/Akt/mTOR pathway, and is intricated in the regulation of autophagy [35].

#### 1.1.3. Immunomodulation

Glioblastoma associated tumor microenvironment is characterized by tumor-associated macrophages, microglia, and immunosuppressive cytokines secreted by tumor cells [36]. Collectively, these factors, such as IL-6, IL-10, prostaglandin-E, and transforming growth factor-beta (TGF-β), inhibits the activation of both the innate and adaptive immune responses, such as inhibition of T lymphocytes and NK cell activity, apoptosis of T cells, the shift of tumor-associated macrophages to an immunosuppressive M2 phenotype, and down-regulation of MHC expression. Moreover, tissue hypoxia up-regulates the immunosuppressive STAT3 pathway, with the downstream synthesis of hypoxia-inducible factor-1 alpha, production of vascular endothelial growth factor, and activation of regulatory T cells, thereby inhibiting the dendritic cells’ response [37]. Plant polysaccharides have shown regulatory effects on the immune system through the stimulation of immune cells and increasing the production of cytokines through the activation of the complement system [23].

#### 1.1.4. Dysregulated Metabolism

Mitochondrial defects are suspected of playing a significant role in tumorigenesis and invasion of tumors, including gliomas. Notably, mitochondrial oxidative phosphorylation (OXPHOS) is defective in tumor cells—a phenomenon known as the Warburg effect. Therefore, tumor cells preferentially rely on glycolytic ATP production rather than on respiration to fulfill cellular energy requirements [38]. This characterizes a slightly distinctive metabolic state of tumor cells, which entails a high consumption of glucose. The difference in energy metabolisms between normal and tumor cells provides a biochemical basis to propose glycolysis as a potentially efficient target for GBM treatment by selectively killing tumor cells through glucose withdrawal or shifting to ketogenic diets [39,40].

Metabolite profiling of U87 glioma cells in mutant lower-grade gliomas show elevated levels of d-2-Hydroxyglutarate 1. Analyses of tumor-associated isocitrate dehydrogenase (IDH) mutations in lower-grade gliomas highlight the significance of associations between epigenetics and metabolism. Additionally, in IDH mutant cells, the production of glutathione is solely reliant on glutaminase-driven glutaminolysis for glutamate biosynthesis, hence IDH mutant glioma cells are more sensitive to oxidative stress than the IDH wild-type cells, thereby suggests glutamine as a primary source of d-2-hydroxyglutarate in tumors with IDH mutations [39,41]. Furthermore, AKT and mTOR are mainly involved in metabolic reprogramming in cancer cells. Akt, acting through AMP-activated protein kinase, is involved in pathways that control glucose and lipid metabolisms by identifying fluctuations in nutrients and extracellular energy levels. Quercetin, a flavonoid, directly binds and suppresses the PI3K activity, which is an upstream regulator of Akt-mTOR signaling [42].

## 2. Chemopreventive Activities of Flavonoids

**Figure 1:**
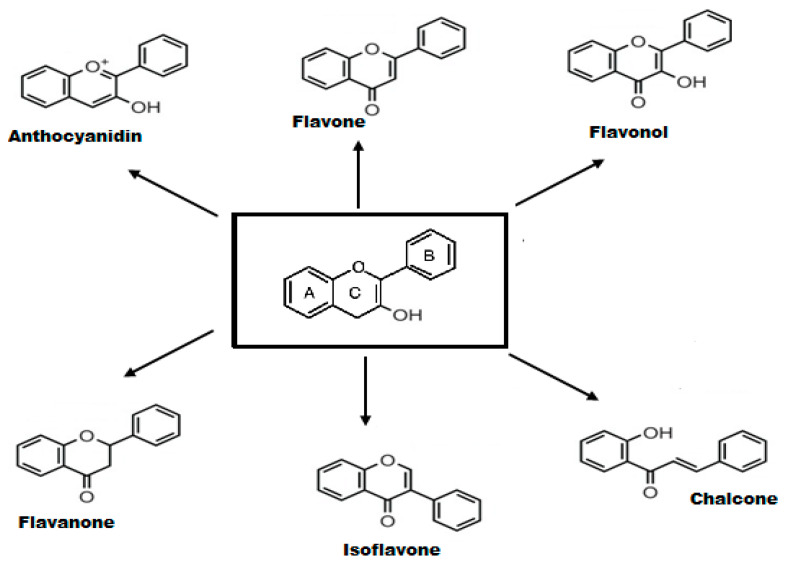
Structure of some common flavonoids.

Flavonoids are phenolic phytochemicals with a wide-ranging structure comprising of two benzene rings designated as A and B, which are connected through the central pyrone ring, designated as C (C6-C3-C6 skeleton) [43]. Based on structural diversity, flavonoids are further categorized into anthocyanidins, flavones, flavanones, flavonols, isoflavones, and chalcones. The structure of main classes of flavonoids is provided in Figure 1. Flavonoids are found either as glycoside derivatives or as free aglycones, that are present in vegetables and fruits [44]. 

Numerous biological activities of flavonoids have been described, such as anti-inflammatory, anti-oxidant, anti-angiogenic, anti-proliferative, and anti-cancer activity [45,46]. Flavonoids regulate multiple signal transduction pathways, such as phosphatidylinositol-3-kinases (Akt), epidermal growth factors, nuclear factor kappa B (NF-kB), mitogen-activated protein kinases (MAPK), p53, Bcl-2 family of proteins, caspases, cyclin-dependent kinases (CDKs), Wnt, Notch, and COX-2, through modulation of certain genes and phosphorylation of the proteins they affect cellular functions [19,47]. 

Flavonoids induced apoptosis is thought to involve the increased generation of ROS with subsequent phosphorylation of p38 MAPK in GBM cells [48]. The phosphorylation of p42 of 44 MAPK is associated with cytoprotective effects [49]. Therefore, increased generation of ROS provides a signal for selective phosphorylation of p38 MAPK and induce apoptosis in GBM cells after flavonoids treatment. Moreover, flavonoids also induce an increase in phosphorylation of JNK1 epigallocatechin gallate (EGCG), selectively trigger apoptosis in human GBM cells, but not in normal astrocytes [48]. 

### 2.1. Bioavailability of Flavonoids

Dietary flavonoids are mainly absorbed through the small bowel, through a glucose transporter; sodium-glucose transport protein 1 (SGLT-1), which transports the glycosidic moieties in enterocytes, where their glycoside bond is cleaved with subsequent conjugation, i.e., methylation, sulfation, or glucoronidation. The unconjugated monomers undergo extensive metabolism in the liver. The bioavailability of flavonoids is further limited as they are substrates of efflux transporters in enterocytes, that may transport them back to the intestinal lumen [50,51].

Flavonoids can pass through the BBB through carrier-mediated trans-cellular transport, trans-cellular, or para-cellular diffusion across the tight junctions of the BBB [52]. However, molecular size is the major determinant in both types of diffusion, which favors small flavonoid molecules like unconjugated monomers. Additionally, passive trans-cellular diffusion is limited to the lipophilicity of the small unconjugated molecules. However, the transport of conjugates and oligomers is aided by certain transporters, such as organic anion-transporting polypeptides (OATPs) [53]. Flavonoids are potential substrates of P-glycoprotein, which is an efflux carrier of substrate molecules from the brain interstitial fluid to the endothelium. Due to unequal perfusion of the brain regions with interstitial fluid, the distribution of flavonoids across different regions of the brain is affected [52,54].

Conclusively, flavonoids have poor bioavailability in their aglycone forms, which is further limited by the presence of the BBB. To overcome this serious problem, different modification strategies, such as pro-drug designing with good bioavailability, pre-methylation of polyphenols to enhance metabolic stability, and developing novel pharmaceutical formulations of flavonoids, e.g., nanoparticles, liposomes to improve bioavailability, efficacy, and specific targeting of tumor cells might be helpful [55,56].

### 2.2. Structure-Activity Relationship of Flavonoids

The existence of a double bond at C2-C3 position, the oxo group at C4, the location of ring B (at C2 or C3), and an open ring C, are the main structural variants that can produce disparity in the anti-cancer properties of flavonoids [43]. For example, a C2-C3 double bond, as well as an unopen ring C (as chalcones have no activity) is needed for flavonoids induced differentiation of cancer cells. In addition, the presence of C2-C3 double bond, an oxo group at C4, and a hydroxyl group at C3, C7, and C4 is required for topoisomerase inhibition of tumor cells. Notably, the substitution of the oxygen atom in ring C by sulfur or nitrogen atoms can cause immense variations in topoisomerase and protein kinase activity [57,58]. 

The type of substituent is also important—for example, the presence of sugar moieties and multiple hydroxyl groups can improve the bioavailability of flavonoids. However, it is noteworthy that glycosides show less activity than their corresponding aglycones in inducing differentiation of tumor cells. Moreover, a slight transformation in the basic structure of the phytochemicals, such as increasing the number of hydroxyl groups, can affect the binding affinity of flavonoids towards a specific protein. For example, the addition of one or two hydroxyl groups in myricetin seemingly increases its binding affinity to MEK protein ligand [42].

In general, variations of substituents at different positions can enormously alter the anti-cancer activity of flavonoids. Such as hydroxylation of flavonoids at C3 and C5 position and methoxylation at C3 and C4 render them more active in regard to cytotoxicity and interactions with tubulin, inducing cell cycle arrest [43].

### 2.3. Chrysin

Chrysin (5,7-dihydroxyflavone) is a 15-carbon skeleton polyphenolic compounds that belong to a class of flavonoids extracted from plants, propolis, honey, the passion flowers (Passiflora caerulea, Passiflora incarnata) and in Oroxylum indicum [59,60]. The pharmacological potential of flavones, as identified in chrysin, is due to the presence of a double bond at C2-C3 in ring C and lack of oxygenation at the C3 position. In contrast to other flavonoids that contain either only one OH group in ring B at C30 position or two hydroxyl groups at C30 and C40, chrysin lacks oxygenation in ring B [61]. Furthermore, diversity in ring A of chrysin results in the formation of various other naturally occurring flavonoids, such as wogonin and baicalein [62]. 

The anti-tumor activity of chrysin is mediated through direct action on multiple molecular targets and modulation of signal transduction of different inflammatory pathways (NFkB, p38/MAPK TBK1, Wnt/β-catenin) and cellular metabolism pathways (AMPK/AKT/ERK/PPAR) [63,64,65,66]. Chrysin also inhibits cell growth of anaplastic thyroid carcinoma through activation of the Notch1 pathway. Moreover, chrysin induces autophagy through elevation of LC3-II levels to increase apoptosis in MCF-7 cancer cell lines [67]. 

Chrysin activity is reported in C6 glioma cells through cell cycle arrest at the G1 phase through the activation of either p38/MAPK signaling that leads to aggregation of p21Waf1/Cip1 protein, or through inhibition of proteasome activity [68]. Several studies demonstrated a possible mechanism of chrysin-induced tumor suppression in GBM cell lines is through inhibition of ErK/Nrf2-signaling-mediated proliferation, invasion, and migration of GBM cells [69]. Chrysin inhibits Nrf2 signaling pathway in anaplastic glioma, specifically, thereby suppressing the Nrf2-mediated expression of hemeoxygenase-1 and NAD(P)H quinine oxidoreductase-1. In addition, chrysin suppresses the phosphorylation of extracellular signal-regulated kinases 1 and 2, but not reduces the phosphorylation of JNK and P38 [69]. Furthermore, chrysin significantly decreases free radicals generation, and enhances the activity of superoxide dismutase, catalase and glutathione peroxidase in the mice model [70]. Chrysin, in combination with silibinin, increases the sensitivity of A172 GBM cell lines towards arsenic trioxide, which shows a high efficacy in treating acute promyelocytic leukemia, but has less sensitivity towards GBM. Chrysin enhances the sensitivity of the A172 cell line through the increased accumulation of arsenic and depletion of cellular glutathione [71]. Furthermore, simultaneous administration of ethanolic extract of propolis (containing chrysin and CAPE) and TMZ results in growth inhibition of U87MG GBM cells by reducing the translocation of NF-κB to the nucleus [72]. Chrysin inhibits TMZ induced autophagy and overexpression of MGMT (both of these factors confer to TMZ resistance towards GBM), thereby sensitizing the GBM cells’ responsiveness to TMZ toxicity. Hence, chrysin serves as a potential anti-tumor agent against GBM in human GBM 8901 cell lines [73]. Chrysin suppresses tumor growth of GL-15 GBM cells by inhibiting cell metabolism, damaging mitochondria, and endoplasmic reticulum, which subsequently leads to apoptosis of GBM cells. Furthermore, chrysin delays the invasion and migration of GBM cells by decreasing the cell surface filopodia-like structures and reducing the protein expression of fibronectin, laminin, and matrix metalloproteinase (MMP-2) [65]. This is similar to another study that analyzed the combination treatment effects of propolis and hypericum perforatum, and reported the pronounced inhibition of invasion and migration of cells by inhibition of MMP-2 and MMP-9 levels in U87 GBM cell lines [74]. 

#### Modified Chrysin

Despite its high therapeutic potential, chrysin has low bioavailability in humans because of its acute metabolism. The enzymes that catalyze the chrysin metabolism, such as M-PST, P-PST, and UGT1A6, possess a significant affinity for chrysin, suggesting limited oral bioavailability of chrysin [75]. Several studies have demonstrated some novel dosage forms of chrysin, such as micelles, liposomes, and nanoparticles, as carriers to enhance the bioavailability of chrysin [76,77,78]. Chrysin-loaded poly (d, l-lactic-co-glycolic acid) PLGA and polyvinyl alcohol have been effectively developed for targeting cancer cells [79,80]. PLGA has been successfully used biocompatible and biodegradable polymer because it is converted to two secondary metabolites, glycolic acid, and lactic acid (both of these molecules are metabolized through the Krebs cycle, which decreases systemic toxicity and enhances therapeutic benefits for efficient drug delivery [81]). For example, chrysin-curcumin in PLGA-PEG (polyethylene glycol) down-regulates cyclin D1 expression and suppresses the proliferation of breast cancer cells [82,83]. Another study demonstrates dose-dependent, potent in vitro anti-cancer potential of modified nanochrysin against SKOV-3 and MCF-7 cancer cells [84]. Furthermore, chrysin loaded bovine serum albumin nanoparticles have been synthesized and conjugated with folic acid to provide a controlled release and enhanced cytotoxic effect on tumor cells [85]. 

Targeted brain delivery of chrysin loaded solid lipid nanoparticles enhances BBB permeation, thereby significantly enhancing chrysin concentration in the brain. A 5 fold increase in the oral bioavailability of chrysin encapsulated in solid lipid nanoparticles has been observed in-vivo as compared to free chrysin [86]. Moreover, chrysin loaded mesoporous silica nanoparticles have been developed and utilized for nose-to-brain delivery, by-passing the BBB. These mesoporous silica nanoparticles demonstrate a pH-dependent release of chrysin with the increased chemical stability of chrysin at a relatively lower pH (5.5), with a 58.2% reduction in cell viability has been observed with olfactory neuroblastoma cells [87]. Examples of some common flavonoids and their activities against GBM are summarized in the Table 1. 

### 2.4. Quercetin

Quercetin is the most abundant flavonoid found in a variety of vegetables, such as broccoli, onion (Allium cepa), Allium fistulosum, Hypericum hericnum, Ocimum sanctum, *and Camellia sinensis*, has long been considered a potent anti-carcinogenic flavonol [92,93,94]. Several structure-activity relationship studies, performed on quercetin related flavonoids, have shown the existence of carbonyl group at C4 and the double bond at C2–C3 position are essential for apoptotic and anti-proliferative activity. However, the substituents required for ring A- and B- still need to be elucidated [95].

Quercetin, considered as a protein tyrosine kinase inhibitor, is reported to inhibit MAPK/ERK 1 and Raf1 kinase activities in cancer [96]. Significant reduction in IL-6-mediated up-regulation of STAT3 is observed in U87 and T98G GBM cells upon quercetin treatment, which subsequently modulates the expression of cyclin D1 and matrix metalloproteinase-2 [97]. Quercetin also sensitizes U87 and U251 glioblastoma cell lines to cytotoxic effects of temozolomide by suppressing the expression of heat shock protein 27; that is implicated in causing resistance to chemotherapy [88]. Furthermore, quercetin also induces mitochondria-mediated apoptosis in the p53 mutant U373MG and T98G resistant GBM cell line as evident by enhanced activities of caspase-9 and caspase-3 and amplified poly ADP ribose polymerase protein cleavage [98]. 

Quercetin significantly reduces protein expression of phosphorylated ERK and Akt in A172 glioma cells, and decreases the expressions of MMp-9 and fibronectin which confers to decreased cell migration and cell viability [99,100]. In addition, quercetin induced apoptosis is linked to decrease expression of survivin (an anti-apoptotic protein) in A172 and U87-MG cells. Survivin degradation subsequently leads to TNF-related apoptosis-inducing ligand-mediated apoptosis [101,102].-Additionally, the inhibition of downstream Wnt/β-catenin signaling transcripts and decrease proliferation of GBM cells has been observed upon isoquercetin treatment, which is a derivative of quercetin [103].

#### Modified Quercetin

Quercetin is a substrate for P-glycoprotein, and therefore, is eliminated from the brain, which decreases its net permeability [54]. Moreover, the poor solubility of quercetin has also limited its therapeutic profile, as its soluble conjugates generally possess very little activity. A prodrug of quercetin QC12 has been synthesized, which is water-soluble, and has undergone phase I clinical trials [104]. Additionally, quercetin analogs comprising of 3- and 5-linked inositol 2-phosphate moieties have been synthesized through a succinate di-ester linkage. A remarkable anti-proliferative activity of a 5-linked analog has been observed against human GBM cells [105]. 

Different nanoparticles loaded with quercetin have been designed to improve the bio-potency and bioavailability of quercetin to increase its anti-tumor activity. The quercetin encapsulated nano-liposomes has significantly enhanced the aqueous solubility and bioavailability of quercetin, thus enhancing the chemotherapeutic efficacy in C6 glioma cells [102]. Interestingly, certain chemically modified polymeric nanocapsules are designed for active and passive targeting of cancer; these are characterized as quercetin carriers. Active targeting of HeLa cells expressing folate receptors has been attained by formulating folic acid PLGA conjugates, using PEG as a spacer in polymeric nanocapsules [106].

### 2.5. Genistein

Genistein (5,7-dihydroxy-3-(4-hydroxyphenyl) chromen-4-one) is a soy isoflavone, obtained abundantly from the Leguminosae family, such as Lupinus spp., Glycine max, Pueraria lobata, and Psoralea corylifolia [107,108]. It is a phytoestrogen and has demonstrated chemopreventive potential in hormonal cancers, such as breast and prostate cancers [89,109]. The anti-neoplastic potential of genistein in non-hormonal cancers, such as colon carcinoma, has also been reported. Genistein-mediated inhibition of NF-κB expression through the down-regulation of Akt, is the main mechanism responsible for apoptosis in human prostate cancer [19,110]. Moreover, reduced expression of Bcl-2 and increased expression of Bax, caspase-3, and -9, is seen upon genistein treatment in vitro. Additionally, genistein down-regulates MMP-9 expression in A549 cell lines and MMP-2 expression in human prostate cancer, thereby preventing invasion and metastasis of cancer cells [63]. Furthermore, genistein reduces the expression of apyrimidinic (AP) endonuclease 1 (APE1) in prostate cancer cells. APE1 is an enzyme tangled in the DNA base excision repair, and its escalated levels are associated with resistance to chemotherapy [111]. Genistein is also a potent inhibitor of protein tyrosine kinase, consequently inactivating the tyrosine kinase-mediated downstream signaling mechanisms [112]. Additionally, the induction of autophagy is thought to be involved in the chemopreventive potential of genistein, against different tumor types, such as lung and breast cancers [22]. 

A combination treatment of rapamycin with genistein demonstrates inhibition of phosphorylation of Akt and its downstream mTOR in U87 human GBM cells [113]. Genistein is reported to prevent the growth of medulloblastoma and GBM cell lines with diverse TP53 mutations through G1 and G2/M cell cycle arrest. Interestingly, genistein exhibits growth arrest in only radiosensitive glioblastoma cells, such as A172 and ONS76, and not in radio-resistant cells, such as KNS60 and U251MG, which is suggestive of the fact that genistein exhibits chemopreventive potential rather than cytotoxic effects and it could be a potential candidate for combination chemotherapy [90]. Similarly, another study reported the synergistic growth inhibitory effects of genistein in combination with carmustine (BCNU) in U87 and C6 glioma cell lines [114]. It has also been revealed to increase the efficacy of other chemotherapeutics (tamoxifen, carboplatin,) and certain other flavonoids, such as quercetin and epigallocatechin gallate [115]. Genistein induces cytotoxicity in LN18, T98G, and LN308 GBM cell lines through the inhibition of topoisomerase II and cell cycle arrest at the G2/M phase through the up-regulation of CDK inhibitors (tumor suppressor genes), such as p21, in these GBM cell lines [116]. Genistein also inhibits telomerase activity by down-regulating the transcriptional activity of telomerase reverse transcriptase, which encodes the catalytic component of telomerase in GBM and neuroblastoma cell lines [90]. Inhibitory effects of genistein on the invasion of tumor cells are evident by inhibiting tyrosine kinase Epidermal growth factor receptor in a co-culture GBM model and also inhibiting urokinase plasminogen activator, which in turn functions in a range of events of the metastatic cascade [113]. 

#### Modified Genistein

Currently, different antibody conjugates of genistein, such as B43-genistein and epidermal growth factor-genistein, are in clinical progress for the amelioration of acute lymphoblastic leukemia and breast cancer, respectively [117,118]. A study (conducted to evaluate the different potencies of multi-compartmental nanoparticles containing paclitaxel and genistein in a Ehrlich ascites tumor model, in swiss mice) found 11% of tumor inhibition with paclitaxel and 44% of tumor inhibition with genistein [119]. Moreover, genistein loaded titanite nanotubes have been designed for controlled drug release and increase cellular uptake of genistein in U87-MG human glioblastoma with promising efficient anti-tumor activity [120]. In another study, genistein-loaded liposomes with an asolectin base has been reported to augment the in vitro anti-tumor effect of genistein on C6 glioma cells by decreasing glioma cell viability, but does not reduce the viability of the normal astrocytes [121]. Moreover, liposomal loaded genistein has exerted anti-tumor effects at a dose lower than the free genistein, thereby increasing its efficacy [121,122]. A recent study described the anti-oxidant and anti-glioma effects of genistein in genistein loaded dimiristoyl-phosphatidyl choline liposomal drug delivery, which decreases the viability of C6 glioma cells up to 80% and also increased the anti-oxidant effects of genistein [123]. 

### 2.6. Epigallocatechin Gallate

Epigallocatechin gallate (EGCG), a flavonoid catechin isolated from dried leaves of the plant Camellia sinensis, has been broadly explored for its chemopreventive and chemo-sensitizing activities in many malignant cancers [124,125]. EGCG regulates the cell transformation of MCF-7 and HeLa cells through inhibition of insulin-like growth factor 1 receptor, which mediates insulin signaling in conjunction with insulin receptors, and is involved in the tumorigenesis of breast and lung carcinoma [42]. Moreover, EGCG is implicated in down-regulating Wnt signaling in breast cancer cells and up-regulating p53 transcription in LNCaP prostate cancer cells [5]. EGCG inhibits the protein expressions of telomerase, and also down-regulates certain inflammatory signaling cascades, such as NFkB, platelet-derived growth factor receptor (PDGF), and IGF-1R in glioma cells [91].

A recent study described anti-glioblastoma effects of EGCG on U87 MG glioblastoma cell line enriched with Glioma Stem-Like Cells (GSLCs); EGCG treatment profoundly reduced the neurosphere formation and migration of tumor cells [126]. Moreover, EGCG down-regulates P-glycoprotein in vitro that results in the sensitization of glioma stem cells to temozolomide [91,127]. EGCG significantly down-regulated Bcl-2 expression and Akt phosphorylation, and increased Bax expression and PARP cleavage, thereby inducing apoptosis in GSLCs [126]. (Similarly, EGCG enhances apoptosis by inducing caspase -9, -3 and c-Jun N-terminal kinase 1 expression and reducing mitochondrial membrane potential in T98G and U87MG human GBM cells [128]. 

There is significant evidence that EGCG enhances the sensitivity of cancer cells to chemotherapeutic drugs (tamoxifen, cisplatin) by suppressing telomerase in 1321N1 and U87-MG cell lines and augments sensitivity of GBM U87 and U251 cells to temozolomide [10,129]. Moreover, EGCG down-regulates glucose-regulated protein 78, a main pro-survival element of the endoplasmic reticulum stress response system, which is the main contributor of cell resistance to temozolomide [130]. Additionally, EGCG inhibits invasiveness in glioma cells by down-regulating matrix metalloproteinases (MMP-2 and MMP-9) expression, and prevents their proliferation by regulating the MAPK signaling [131]. Furthermore, EGCG inhibits the proliferation of U87 cell lines and retaliate the effects of survivin (anti-apoptotic protein) overexpression that otherwise shows resistance to ionizing radiations. Moreover, EGCG strengthens the effects of ionizing radiation in human brain microvascular endothelial cells by enhancing the expressions of CDK inhibitors (p21, p27) and stimulation of cell necrosis in the perivascular niches that consist of therapy-resistant glioblastoma stem cells [132,133]. 

#### Modified EGCG

A series of EGCG analogs (represented as MST-199) have been synthesized wherein the ester linkage is substituted with an amide linkage to enhance the stability. The anti-telomerase activity of these derivatives is related to the presence of 3, 4-hydroxy groups on both of the B and D rings [134]. 

**Figure 2:**
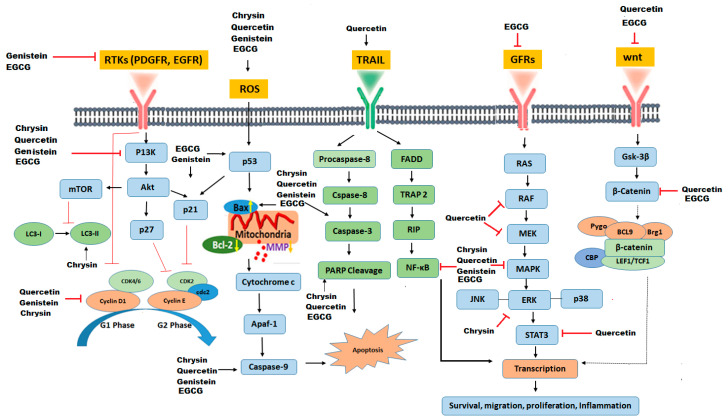
Signal transduction pathways targeted by flavonoids in glioblastoma. Such as survival factors RTK (PDGFR, platelet-derived growth factors; EGFR, epidermal growth factor receptors), TNF/TRAIL death receptors, growth factor receptors and frizzled Wnt receptor.

Although EGCG’s stability in liposomes and nanostructured lipid carriers is considerably high [120], premature deterioration of EGCG can be further prevented by nanoparticles [135]. Nanoparticles loaded with EGCG show a sustained-release pattern that reduces the dose and frequency of treatment, as well as the adverse side effects [136]. In addition, targeted delivery of EGCG to cancer cells can be improved by integrating specific ligands on the surface of EGCG nanoparticles, such as targeted delivery of EGCG to prostate cancer cells expressing prostate-specific membrane antigen has been successfully devised [137]. Moreover, epigallocatechin-3-gallate loaded hyaluronic acid nanoparticles specifically targets CD44 (multifunctional cell surface glycoprotein associated with migration and proliferation of cells), binds to CD44 receptors, causes cell cycle arrest at the G2/M phase, and increases apoptosis of prostate cancer cells [138]. In another study, EGCG derivatives have been used for carrying and delivering herceptin to breast cancer cells by making micellar nano-complexes. Conclusively, these nano-complexes effectively decreases cell viability and suppresses tumor growth in breast cancer [139]. Some common pathways targeted by flavoinds in amelioration of GBM are mentioned in Figure 2.

## 3. Chemopreventive Activities of Polysaccharides

Plants are a wide source of a variety of polysaccharides that have been demonstrated for several biological properties, such as immunomodulatory, anti-tumor, anti-oxidant, anti-inflammatory, anti-angiogenic, and hematopoietic effects [140]. 

Conventional cancer therapies, such as radiotherapy and chemotherapy, are associated with severe adverse effects on the immune system [141]. Moreover, tumor cells can evade the immune surveillance, proliferate, and survive by a variety of mechanisms. Hence, the disease and treatment are immuno-suppressive. This observation paved the way for the development of several strategies to potentiate anti-tumor immune cell response to counter the ability of tumor immune evasion [142,143]. In this context, the immuno-stimulatory agents can represent an ideal alternative for immune modulation and can enhance the efficacy of chemotherapeutics as adjuncts. Polysaccharides are also referred to as biological response modifiers because of their immunomodulatory effects, and are currently among the most active areas of immuno-pharmacology research [23]. 

The possible immunomodulatory mechanism of action of botanical polysaccharides is likely to involve specific interactions with various cell surface receptors, such as Dectin-1, lactosyl ceramide, Complement receptor-3 (CR-3), selected scavenger receptors (SR), TLR-4 and TLR-2. Polysaccharides binding to these receptors leads to activation and signal transduction of T lymphocytes, NF-kB and MAPK, subsequently increasing the release of certain chemical mediators (such as cytokines and colony-stimulating factors) and activation and stimulation of immune cells, such as lymphocytes, dendritic cells, cytotoxic macrophages, and natural killer cells [144,145,146], which inhibits proliferation of cancer cell through either direct cytotoxicity, cell cycle arrest and apoptosis of cancer cells, or through inhibition of angiogenesis and metastasis of cancer cells [147,148]. 

**Figure 3:**
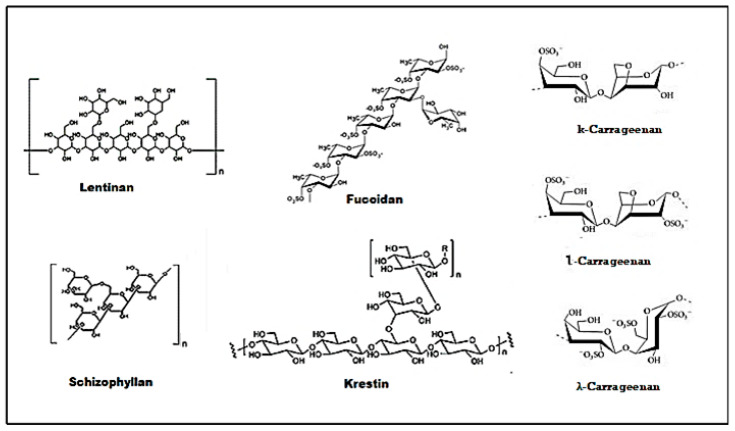
Structure of some common polysaccharides.

Amongst the wide variety of polysaccharides with potential anti-tumor activities, lentinan, schizophyllan, krestin, fucoidan, and carrageenan deserved much attention. For many years these polysaccharides have been effectively used in the treatment of cancer, and their mechanisms of action are well established. A small number of these polysaccharides have been proceeded to clinical trials and for licensing as cancer treatments [149]. The structure of some common polysaccharides are provided in Figure 3. 

### 3.1. Structure-Activity Relationship of Polysaccharides

The potency of anti-cancer polysaccharides appears to be correlated to their molecular weights, degree of branching, aqueous solubility, and by three-dimensional structure [147]. 

Molecular weight is the major determinant of anti-tumor activity of polysaccharides. Polysaccharides with molecular weights ranging between 20 kDa to 500 kDa have demonstrated better bioactivities because polysaccharide molecules in this range possess a relatively complex structure and have good water-solubility [146]. For example, Lichenan, a polysaccharide with a molecular weight ranging between 20 kDa to 62 kDa has very good anti-tumor activity. Similarly, lentinan having a molecular weight of 500 kDa, possess very potent anti-tumor activity is used in clinical medicine. However, polysaccharides of molecular weight deviating from this range have reduced bioactivities [150]. 

The main chain saccharide unit in polysaccharides is also an important determinant of anti-tumor potency. Studies have reported that (1→3) and (1→6)-glycosidic bonds on glucan chains in polysaccharides play a crucial role in determining the anti-tumor activity. For example, lentinan has main-chains of (1→3)-β-d-glcucopyronysyl along with many (1→6)-β-glucosyl side branches have a conspicuous anti-tumor capacity [146]. In general, polysaccharides have two molecular configurations—α and β. Interestingly polysaccharides with an α-configuration have poor activity, while those with β-configurations have strong bioactivity. For instance, bioactivity is much better in (1→3)-β-glcucopyronysyl than (1→3)-α-glcucopyronysyl. Such as lentinan with (1→3)-β-d-glcucopyronysyl main-chain has potent ant-tumor activity [151]. 

The helical conformation is considered to play a significant role in improving immuno-potentiating activity—mostly polysaccharides in triple strand helical chain structure exhibit stronger anti-tumor activity compared to random coils or lines [152]. For example, triple-helical lentinan exhibited potent anti-tumor effects in mice with an inhibition rate of 49.5%, which is close to that of a reference anti-cancer drug. However, this bioactivity promptly decreases upon transformation to a single-flexible chain conformation, thereby displaying the strong relation between the anti-tumor action and the triple-helical conformation of lentinan [151,153].

Another important determinant for the mechanism of anti-tumor activity is the distribution of branch units beside the main backbone chain. The anti-tumor activity of polysaccharides is linked to an optimum degree of the branch (DB) of side-chains, i.e., the number of side chains in every saccharide unit. Hence, to achieve a certain level of bioactivity, the polysaccharide molecule must reach a certain DB. The DB of side chains should not be too high or too low for bioactivity. As, too high DB indicates increase bulk of polysaccharide molecule, which can decrease water-solubility, thus lowering the polysaccharide bioactivity. Besides, too low DB polysaccharides have no anti-tumor activity [154,155]. 

Generally, the most active polysaccharide polymers possess a DB ranging from 20% to 33%. Lentinan has a DB of 40%. A recent study showing the association between branching and biological activity revealed that the de-branching of lentinan enhanced its biological proficiency. Maximum immuno-modulating and anti-tumor activities were attained with a DB of 32% [156]. However, pachyman a polysaccharide with DB of 1.5% to 2% has no anti-tumor activity, although it has the same configuration as lentinan [157]. 

**Figure 4:**
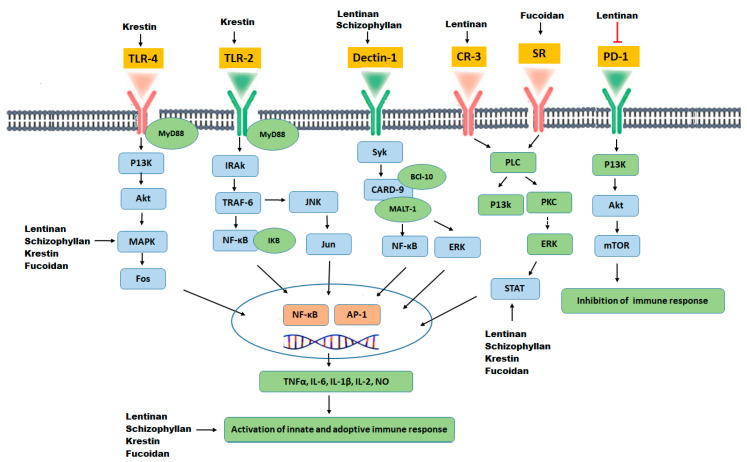
Immune regulation by plant polysaccharides through cell surface receptors present on macrophage and dendritic cells; toll-like receptors (TLR-4, TLR-2), Dectin-1, complement receptor 3 (CR3), scavenger receptor (SR) and programmed cell death receptor (PD-1), present on T lymphocytes, Mammalian target of rapamycin (mTOR), TNF receptor-associated factor 6 (TRAF6). IL-1R-associated kinase (IRAK), Spleen tyrosine kinase (Syk), Caspase recruitment domain-containing protein 9 (CARD-9).

The bioactivity of polysaccharides that possess anti-tumor activity and immuno-potentiation can be increased by certain chemical modifications. Such as sulfation, methylation, carboxylation, and hydroxylation, which results in more effective anti-tumor activities [146,158,159]. Characteristically, a series of sulfated or carboxymethylated polysaccharides derivatives can improve anti-tumor activities [159]. For example, sulfated fucoidan has potent anti-tumor activity in many different types of cancers [160]. Furthermore, chemically modified methylated or ethylated derivatives of polysaccharides has shown the potential to suppress tumor development. For example, and formyl methylated and amino ethylated derivatives from schizophyllan has enhanced the production of tumor regression factors, as well as soluble cytotoxic factors, thereby enhancing anti-tumor activities in comparison with native schizophyllan. Similarly, hydroxylated derivatives of schizophyllan have stimulated macrophages induced production of NO and TNF-α as compared to native schizophyllan [21]. A schematic overview of the immunomodulatory action of polysaccharides is explained in Figure 4. 

### 3.2. Lentinan 

Lentinan (LNT) is a medicinal polysaccharide purified from Lentinus edodes, an edible and medicinal mushroom, known to have potent anti-tumor activity through stimulation of the human immune system. The main chain of lentinan is formed of β (1→3)-d-glucan with two side chains of β-(1→6)-d-glucopyranosyl at every five β-(1→3)-d-glucopyranosyl repeating units [152,161] 

Lentinan exerts its immunomodulatory action by binding to immune cell surface receptors, such as Dectin-1 and CR3, and stimulates the release of cytokines like TNF-α, IL-1α, IL-1β, IL-1, IL-2, IL-3, IL-6, IL-8, interferon (IFN), and colony-stimulating factor—subsequently triggering the maturation, differentiation, and proliferation of immune cells for host defense mechanism [162]. Lentinan has been demonstrated as an adjuvant that enhances NK cells and T cells activity, and it shifts the balance of Th1/2 towards Th1 by increasing the production of IL-12 [163]. Lentinan treatment inhibits prostaglandin synthesis in patients with stomach cancer, which slows down the differentiation of T lymphocytes and inhibits the activity of T regulatory cells. Lentinan also increases activation of cytotoxic T lymphocytes level in the spleen, stimulates B-lymphocyte proliferation, and enhances antibody-dependent cell-mediated cytotoxicity [21,149,164]. 

In China, lentinan has been extensively employed as a therapeutic agent in the treatment of malignant tumors for almost 20 years [165]. Lentinan has been employed as an adjuvant for the clinical treatment of gastric cancer with negligible side-effects. Furthermore, lentinan alleviates toxicities of chemotherapeutic agents, thereby improving the efficacy of chemotherapy [166,167]. Lentinan is described to down-regulate the expression of hTERT genes by suppressing C-myc in DLD-1 cancer cells, thereby inhibiting the telomerase activity in cancer cells [168]. A study evaluated the anti-angiogenic effects of lentinan on tumor growth showed that lentinan treatment-induced IFNγ production, which contributes to tumor vascular growth inhibition in LAP0297 lung cancer cells. Furthermore, expression of TNFα, IFNγ, CXCL9, and TIMP1 (intrinsic angiostatic factors) genes were up-regulated upon long-term treatment with lentinan, thereby inhibiting angiogenesis [166]. 

A novel mechanism of lentinan for the treatment of gastric cancer has been demonstrated through inhibition of programmed cell death ligand 1 (PD-L1), which binds to programmed cell death receptor 1 (PD-1) expressed on activated T lymphocytes [169]. This protects tumor cells from T cell lysis through activation of downstream inhibitory signaling of the T cell antigen receptors [170]. PTEN inactivation leads to the up-regulation of phosphatidylinositol-3 kinase (PI3K)/AKT pathway, which may be associated with intrinsic induction of PD-L1 ([171,172]. Additionally, the activation of several pathways involved in proliferation and cell survival, such as MAPK, NF-κβ, and STAT3, are implicated in regulating PDL-1 expression [173,174]. Synergistic effects of lentinan have been shown in combination with oxaliplatin in the inhibition of NF-kB, STAT3, and survivin expression in HepG2 cell lines in mice with H22 tumor [175]. In human osteosarcoma cells, lentinan treatment induces autophagy and apoptosis through inhibition of the MAPK/ERK signaling by up-regulation of miR-340 in MG63 cells. LNT decreases cyclin D1 levels and increases caspase-3, -9, LC3B-II/LC3B-I, and Beclin-1 levels [176]. 

Lentinan significantly inhibits the growth of rat C6 glioma cell lines, induces apoptosis, blocks cell cycle, increases the proportion of cells in the G0/G1 phase, and lowers the proportion of S-phase cells [177]. 

#### Modified Lentinan

Intravenous administration of lentinan increases the intestinal mucosal immunity in patients with gastric cancer. However, oral administration of lentinan is not effective because of the large particle size (100–200 µm) that hinders the absorption from intestinal mucosa [178]. However, an oral dosage form of superfine dispersed lentinan (SDL) has been designed by nano-technology procedures with 0.2 µm particles of lentinan dispersed in aqueous solutions that can permeate the mucosal barrier, is now available for clinical use [179]. SDL is reported to potentiate the mucosal immunity of the intestine. A study reported the clinical efficacy and safety of SDL in patients with advanced colorectal cancer: SDL suppressed the adverse side effects of chemotherapy and has promising binding affinity for peripheral blood CD14+ C monocytes to lentinan, therefore improving patients quality of life who are subjected to SDL treatment [178]. 

### 3.3. Schizophyllan

Schizophyllan is obtained from Shizophyllum commune, a species of mushroom, which is an extensively studied polysaccharide with immune-modulating anti-tumor properties [180,181]. Schizophyllan with a molecular weight of ~450 k Da, comprising of a β-(1→3)-d-glucopyranosyl main chain and β-(1→6)-d-glucopyranosyl side chains at every third monomer of the main chain [182]. Due to its reversible coiled-helical conformation, it generates a very firm triple-helical structure in water and in other aqueous solutions [183]. 

The mechanism of schizophyllan-mediated anti-tumor action is through binding to the Dectin-1 receptor with subsequent stimulation of the immune system [151]. Schizophyllan as a biological response modifier enhances the production of lymphocytes and macrophages, activates phagocytes, and stimulates the release of immune cytokines, such as IL-1, IL-2, IL-3, IL-6, IL-8, TNF-α, and IFN-γ, which further activates cytotoxic T cells, natural killer cells, lymphoid cells, and bone marrow cells [21]. Moreover, schizophyllan has been extensively employed as a chemotherapeutic agent along with radiation therapy in Japan. It has been approved for clinical use in Japan for the amelioration of solid sarcoma, increases the prognosis of patients with gastric cancer, head and neck cancers, and stage II cervical cancers [184]. Moreover, it improves prognosis and decreased recurrence for stage II cervical cancer, and it is more effective when injected directly to the tumor mass, suggesting direct cytotoxic effects to tumor cells [148]. Furthermore, schizophyllan induced apoptosis is thought to be mediated through increased caspase-3 levels in 7, 12 Dimethyl Benz (a) anthracene-induced hepatocellular carcinomas [185]. 

Recently, it has been described that schizophyllan treatment causes significant growth inhibition of the rat CNS-1 glioma cells through p53-mediated inhibition of cell cycle and apoptosis. Moreover, an increased percentage of cells in the G0/G1 phase, and a diminished percentage of cells in the S-phase have been described [168]. Schizophyllan improves tyrosine 15 phosphorylation by deactivating CDK1 and subsequently enhancing the proportion of cells in the G2/M phase, as well as a reduction in G1 phase cells [21].

#### Modified Schizophyllan

Recently, a novel designed nano-gel of schizophyllan biopolymer has made it a possibility for designing an optimal drug delivery system, due to the tunable size, the biocompatibility, and the swelling properties of schizophyllan-based nano gels [186]. Moreover, novel folate-conjugated schizophyllan show specific affinity towards folate binding proteins and acts as a non-cytotoxic cancer-targeting antisense carrier that is attributed to effective antisense activity in cancer cells [187]. 

### 3.4. Krestin

Krestin, also known as polysaccharide k, is a protein-bound glycan derived from cultured mycelium Coriolus versicolor. Krestin (molecular weight; 1 × 105 Da) contains glucose as the main monosaccharide unit, having a β-(1-4) linkage main chain and a side chain as β-(1-3) and β-(1-6) that is covalently connected to protein constituent through β-(1-6) glucose side chain [162,182]. Noteworthy, a high degree of the structural complexity of polysaccharide molecule is suggestive of strong immunomodulatory and anti-tumor activity, as polysaccharides bound to proteins or peptides conferred more potent anti-tumor effects than the corresponding free glucans [151,188]. 

Previously published clinical literature indicates that krestin has long been used for treating acute non-lymphocytic leukemia, lung carcinoma, gastric and colorectal cancers, and primary hepatic cancers [162]. Krestin acts as a selective TLR2 agonist, and activates both innate and adaptive immune systems; the anti-tumor activity of krestin is dependent on cytotoxic T cells and natural killer cells, but not dependent on CD4^+^ T cells. Moreover, krestin has shown no effects on tumor growth inhibition in TLR2 deficient mice, which further strengthens the idea that TLR2 is involved in the anti-tumor effects of krestin, with the subsequent activation of an immune response [189]. In another study, krestin treatment dose-dependently up-regulated NF-kB expression in HEK cells upon transfection with TLR2 [190]. In addition, krestin also binds to toll-like receptor 4, and increased the release of inflammatory cytokines, such as IL-6 and TNF-α [191]. Some genetic studies suggested that krestin activates leukocytes and lymphocyte-activated killer cells through the up-regulation of key immune cytokines. Furthermore, the anti-metastatic activity of krestin is discovered, which might be ascribed to its potential to prevent metalloproteinases and other enzymes involved in the metastasis of tumor cells [21]. Another study demonstrated the adjuvant chemotherapeutic effects of krestin in combination with 5-flourouracil in colon carcinoma through inhibition of NF-kB and subsequent inhibition of oncogenic β-catenin activation (that causes inhibition to apoptosis of cancer cells) [192]. 

Recent research suggests that krestin treatment before and after surgery improves the survival of GBM patients to four years by effectively enhancing the immune states of patients. Moreover, the humoral immune parameters, such as elevated IgG and IgM serum levels, are also associated with the increased prognosis of patients with GBM. In another follow-up study, krestin efficacy was evaluated in patients with GBM when co-administered with vincristine and nimsustine along with synchronized radiotherapy postoperatively. The connotation between the histological malignancy and the prognosis was investigated—the survival rate improved to 12.3% for three years. Examination of combined effects of krestin and vincristine on GBM tumor implanted nude mice showed PSK and VCR against GBM were more potent when administered intraperitoneally as compared to the local administration to tumor mass [193]. A clinical study using a combination chemotherapy regimen, named as AUFRAP for the treatment of malignant gliomas, this combination comprises of ACNU (nimustine), UFT (uracil + tegafur), radiation, vitamin A and Polysaccharide K, reported shrinkage of the tumors mass and a recurrence-free survival of patients [194].

#### Modified Krestin

Krestin based zein microspheres have been designed successfully for cancer immunotherapy, and a considerable increase in drug release (70–80%) was observed. These microspheres were prepared as a mono-dispersed system of glutaraldehyde, dl-camphorsulfonic acid, and polyvinylpyrrolidone with zein as a carrier matrix, sonicated to generate microspheres with a particle size of less than 1 micrometer, which would be an appropriate size for phagocytosis by macrophages [195]. A summary of some common polysaccharides and their activities in GBM are described in Table 2. 

### 3.5. Fucoidan 

Fucoidan, a sulfated polysaccharide primarily derived from brown seaweed, such as Saccharina japonicus and Undaria pinnatifida, possess potent anti-tumor and anti-angiogenic activities [199,200,201]. Fucoidan is structurally similar to heparin with a significant proportion of l-fucose, sulfated ester groups, and a lesser percentage of glucuronic acid, d-galactose, d-xylose, and d-mannose [154]. The low molecular weight fucoidans principally contain fucose residues and a huge proportion of sulfate groups, with potential anti-cancer effects than high molecular weight hetero fucans with a minimum degree of sulfation [202,203]. 

The anti-neoplastic potential of fucoidan is described through immunomodulatory, anti-inflammatory, anti-angiogenic, anti-proliferative, and pro-apoptotic activities in many different types of cancer cell lines and animal studies [204,205,206,207]. The immunomodulatory effects of fucoidan are demonstrated through the activation of natural killer cells and dendritic-cell-mediated activation of cytotoxic T cells. In the spleen, an up-regulation of maturation markers has been observed in dendritic cells along with the increased release of cytokines (IL-6, IL-12, and TNF-α), Th1 driven immune response, and cytotoxic T cell activation [160]. Some potential molecular targets for fucoidan includes inhibition of nuclear factor-kappa β (NF*κ*β), activator protein-1 (AP-1), reduction of the β-catenin, activation of GSK-3β followed by reduction of cyclin D1, c-myc, c-jun, and c-fos transcription. Certain apoptosis targets include p38, Bax, Bcl-xL, caspase-3, and caspase-7, JNK, ERK1/2, and PI3K-Akt-mTOR signaling. Fucoidan inhibits invasion and development of tubules through inhibition of MMP-2, -9 activity and suppressing VEGF expression in cancer cell lines [42]. These pathways are also implicated in glioblastoma, thereby increasing the likelihood that fucoidan would also be successful in the treatment of glioblastoma. Liao et al., evaluated the differentiation-inducing effects of oligo-fucoidan in U87MG cells and GBM8401 cells. Results showed that oligo-fucoidan substantially suppressed the proliferation of MG cells and inhibited the protein expression of DNA methyl-transferases (DNMT1, 3A, and 3B) followed by up-regulation of differentiation markers (GFAP, MBP, MAP2, and OLIG2) in both GBM8401 and U87MG cell lines, as well as a decrease in methylation of p21 has also been observed with DNMT3B target gene [196]. 

Fucoidan is described to exert detoxification and anti-carcinogenic effects in human hepG2 hepatoblastoma and rat C6 glioma cells through inhibition of the propagation of tumor cells and up-regulation of 7-*O*-de-methylase (MROD), cytochrome C, and phase II enzymes [208]. Fucoidan induced suppression of inducible NO synthase expression and decreased production of nitric oxide (NO) is determined by the up-regulation of JAK/STAT, MAPK, p38, IRF-1, and AP-1 pathways, which depends on the increased expression of the scavenger receptor B1 in the C6 glioma cell stimulated by TNF-α-and IFN [209,210].

The anti-angiogenic effect of fucoidan is also explored in GBM cells and monocytes, as both the GBM cells and monocytes are considered to have the tendency to release soluble fms-like tyrosine kinase-1 (sFlt-1), that is involved in sequestration of VEGF [211]. An increase in sFlt-1 levels was documented in fucoidan-treated glioma cells where angiogenesis was repressed by elevations of the sFlt-1 level [212]. For gliomas, the sFlt-1 level is reported in correlation with VEGF, microvascular density, tumor malignancy, and prognosis [213,214]. Fucoidan inhibited angiogenesis by VEGF down-regulation and was attributed in T98G or THP1cells to elevated sFlt-1. In addition, fucoidan treatment causes a tenfold increase in sFlt-1 levels in T98G cells, but in THP1 cells, there was a seven-fold increase in sFlt-1 level [212]. Fucoidan limits the development of tubular structures in cell lines T98 G and THP1 in endothelial cells [212]. 

#### Modified Fucoidan 

Fucoidan-based nanoparticles (Fucoidan-PEG-hydrazide NPs) directly targeting P-selectin have more effective effects on apoptosis in a cell line of osteosarcoma compared to native fucoidan. More potent effects of fucoidan nanoparticles were verified in vivo using a model for xenograft osteosarcoma [215]. In addition, increased permeation of nanoparticles fucoidan has been documented in Caco-2 cell transport studies, suggesting an increased bioavailability of fucoidan in nanoparticles [216]. 

Another research tested the effects of nanoparticles of fucoidan-coated manganese dioxide in radio-resistant pancreatic cancer. These fucoidan nanoparticles reversed the hypoxia-induced radio-resistance, through enhancing the persistence of clonogenic tumor cells and increasing apoptosis in responses to radiation therapy. They also suppressed tumor angiogenesis by inhibiting the phosphorylation of vascular endothelial growth factor receptor 2 (VEGFR2) and CD31, thereby delaying tumor development in a mouse model of BxPC3 xenograft [217]. 

### 3.6. Carrageenan 

Carrageenans are a group of highly sulfated polysaccharide diversely found in marine red seaweeds; different subtypes of carrageenan are obtained from distinct species of Gigartinales (Rhodophyta) [218]. Structurally, carrageenans are composed of linear chains of d-galactopyranosyl units linked via alternated (1 → 3)-β-d-and (1 → 4)-α-d-glucoside, in which sugar units have one or two sulfate groups, with a molecular weight ranging from 30 and 5000 kDa; however, the average molecular weight of carrageenan is 200 and 800 kDa upon extraction [210]. Carrageenans are commonly divided into six basic types: Iota (i)-, Kappa (j)-, Lambda (k)-, Mu (l)-, Nu (m)-, and Theta (h)-carrageenan [219]. However, commercially important carrageenans are categorized as kappa (k-), iota (i-), and lambda (λ-) carrageenan, which differ in the number and position of the sulfate groups. Kappa (κ)-carrageenan is mostly extracted from the tropical seaweed Kappaphycus alvarezii, while iota (ι)-carrageenan is mainly acquired from Eucheuma denticulatum and lambda (λ)-carrageenan is obtained from various species from the genera Gigartina and Chondrus [218].

Carrageenan’s anti-tumor potential has been studied recently, with several in vitro and in vivo models proposing an anti-proliferative action against tumor cells [220,221]. The immuno-modulatory effect of λ-Carrageenan suggests λ-carrageenan could be an efficient adjuvant in cancer immunotherapy for inhibiting cell growth and to enhance tumor immune response [218,222]. Carrageenan has the potential to arrest cell cycle at the G1, G2, or S phase [223,224]. A previous study demonstrated significant anti-proliferative effects of carrageenan on HeLa cervical cancer cells by arresting the cell cycle in specific phases, thus delaying the cell cycle progression [223]. Furthermore, different carrageenan subtypes, such as λ-CO and k-CO, exhibit different effects on the cell cycle in HeLa cells; k-CO display growth inhibitory effects by delaying the cell cycle in the G2/M phase. On the other hand, λ-CO stalls the cell cycle in both G1 and G2/M phases, resulting in a longer cell cycle as compared to untreated controls [223]. Additionally, λ-CO suppresses the ability of the cell to divide, demonstrating a strong anti-proliferative effect [223]. 

Recent studies suggest that carrageenan inhibit heparin-binding growth factors, such as basic fibroblast growth factor, platelet-derived growth factor, and transforming growth factor 3 and display anti-proliferative activity against several tumor lines that require heparin-binding growth factors for tumorigenesis, such as prostate cancer cell lines, breast cancer cells and lung carcinoma [225]. In addition, carrageenan inhibits the neovascularization of tumor mass by inhibiting certain heparin-binding angiogenic factors [225]. Anti-proliferative effects of κ-carrageenan on MCF7 and HT-29 cancer cells is linked to mitochondria-related apoptotic pathway caused by direct and indirect caspase-3 activation [226]. Moreover, λ-carrageenan inhibits the proliferation of MDA-MB-231 cells by up-regulating pro-apoptotic caspase-8, caspase-9, and caspase-3, which ultimately results in increased levels of active caspase-3 protein. Furthermore, λ-carrageenan also disrupts mitochondrial function by altering the expression of bax/bcl-2 ratio, which is considered an important element in apoptosis induction [227]. Carrageenans extracted from *Gigartina pistillata* in two different fractions is shown to have significant anti-tumor potential against colorectal cancer stem-like cells, with greater efficacy of tetrasporophyte carrageenan (λ/ξ) possibly due to higher sulfate contents as compared to female gametophyte carrageenan (κ/ι) with exclusively fewer sulfate contents [218]. 

Co-administration of λ-carrageenan with chemotherapeutic 5-flourouracil potentiates growth inhibition in xenograft tumors S180 and H22 with cell viability of 51.73% and lead to a stronger anti-tumor effect exerted by the drug [198]. Furthermore, chemically modified derivatives obtained by sulfation, acetylation, and phosphorylation significantly increase anti-tumor activity of κ-oligo carrageenans in mice tumor xenografts S180 model. Anti-tumor effects of sulfated and phosphorylated derivatives of κ-oligo carrageenans are similar to the effects of ftorafur, which is a potent chemotherapeutic agent [198]. A previous study demonstrated promising anti-proliferative activity of sulfated carrageenan obtained from the red alga *Laurencia papillosa* against T98G GBM cell line. This study showed an increase in active caspase-3 protein levels upon carrageen treatment, resulting in an increased apoptosis of T98 GBM cells [228]. 

Novel nanostructured lipid-carrageenan hybrid carriers (NLCCs) have been synthesized for controlled delivery of water-soluble chemotherapeutic agent mitoxantrone hydrochloride (MTO) with sustained-release property has shown 3.5-fold increase in bioavailability and has improved anti-tumor efficacy. The cytotoxicity evaluation indicates that NLCCs can significantly enhance the anti-tumor efficacy against resistant MCF-7 cancer cells through by passing the breast cancer resistant proteins [229]. Gold nanoparticles (AuNPs) implying κ-carrageenan oligosaccharide as a reducing and capping agent show significant cytotoxic activities to HCT-116 and MDA-MB-231 cancer cells [230]. Furthermore, in vitro anti-cancer efficacy of biocompatible ι-carrageenan-γ-maghemite nano-composite has been demonstrated in the human colon cancer cell line by inducing cell apoptosis through the ROS-mediated mitochondrial pathway, along with down-regulation of XIAP and PARP-1 expression, and the up-regulation of expression of caspase-3, Bcl-2, and Bcl-xL [197,231]. 

## 4. Conclusions

Since glioma-genesis is linked to multiple molecular pathways, therefore, to effectively halt tumor progression, a drug that can target multiple deregulated pathways would be ideal. However, there are limitations associated with targeted therapies because tumor cells can develop resistance towards these therapies. Therefore, a combination of targeted therapies could be the solution to the problem. Natural compounds are well known for their diverse molecular mechanisms of action at multiple levels of tumorigenesis. Hence, natural compounds would be ideal as anti-glioblastoma agents, either as monotherapy or in combination with other anti-neoplastic agents to increase their sensitivity towards glioblastoma. However, the foremost challenge in the amelioration of GBM is insufficient drug delivery, due to the presence of BBB. This review highlights the role of certain modified formulations, such as nanoparticles, liposomes, and drug conjugates, to overcome the obstacle of BBB and to improve the efficacy of drug delivery for the treatment of GBM. This review also provides a clear demonstration of the anti-glioblastoma potential of natural compounds, which is indicative of a relatively more effective and safe glioblastoma treatment option.

## 5. Future Directions

As phytochemicals target multiple pathways, therefore using a combination of agents or a multi-targeted therapy that provides additive or synergistic chemotherapeutic effects could be a good option for the treatment of glioblastoma. Administration of a lower active dose of a natural compound could decrease the potential for adverse side effects. However, there exists some ambiguity in defining, which protein is the prime physiological target and which protein is the most desired target. Moreover, only a slight difference in the molecular structure of some compounds can lead to considerable alterations in the target proteins. Therefore, to define candidate targets, an ideal approach would be to carry-out proteomics profiling. Moreover, phytochemicals generally have less specificity towards single target proteins compared to synthetic antagonists, and this could be advantageous for emerging phytochemicals as multiple inhibitors. Keeping in view the relatively low toxicity of phytochemicals and the need for chemotherapeutic agents to be administered over a prolonged period, multi-targeting phytochemicals would be an effective solution for chemoprevention.

## Figures and Tables

**Table 1:** Summary of activities of flavonoids in glioblastoma.

Flavonoid Phytochemical	Structure	Mechanism of Action	References
**Chrysin**	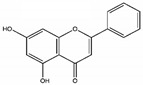	Down-regulation of Wnt, NF-kB, and Akt. Reduction of Erk/Nrf2, modulation of MAPK/ERK, and P38 increase of SOD, CAT. Up-regulation of LC3-II and PARP	[88]
**Genistein**	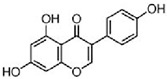	Down-regulates NF-kB and Akt pathways, up-regulation of p53 and p21, inhibition of cyclin B, cyclin D1, TERT expression, activates Notch 1 Signaling pathway	[89,90]
**Quercetin**	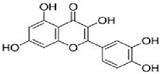	Inhibition of MAPK/(ERK) kinase, (MEK) 1 and Raf1 kinase, STAT3, CDK1, MMP, Akt/P13k pathway. Stimulation of Bid, Bad, Bax, caspase-9, -3 release, Inhibition of Bcl-xL, Bcl-2, and cytochrome c	[69]
**EGCG**	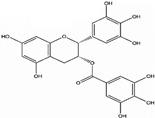	Inhibition of PI3K/Akt pathway, modulation of MAPK, NFκB, Wnt/β-catenin, up-regulation of p53 and p21, G1,S, or G2/M arrest.	[91]

**Table 2:** Molecular targets of polysaccharides in glioblastoma.

Polysaccharide Phytochemical	Molecular Target	References
**Lentinan**	Binding to CR-3 and Dectin-1 receptors, activation of macrophages, natural killer cells, T cells and B cell, Inhibition of T regulatory cells. Cell cycle arrest through the enhanced number of cells in the Go/G1 phase and reduced cells in the S phase.	[166]
**Schizophyllan**	A ligand of the Dectin-1 receptor. Potentiate natural killer cells (NK) and cytotoxic T cells. Inhibition of cell cycle at the Go/G1 and G2/M phase, p53 up-regulation, CDK1 inhibition.	[162]
**Krestin**	A specific TLR-2 agonist, up-regulation of NF-kB and Cytokines (TNF-α, IL-6), enhanced serum IgG, and IgM production. Activation of natural killer cells and lymphocytes activated killer cells.	[168]
**Fucoidan**	Binds specifically to scavenger receptors. Potentiates NK cells, dendritic cells (DC), and T cells. Down-regulated VEGF and elevated sFlt-1. Up-regulation of Myelin Basic Protein (MBP), Glial fibrillary acidic protein (GFAP), Oligodendrocyte transcription factor (OLIG2), and microtubule-associated protein-2 (MAP2). Up-regulation of NF-kB and AP-1	[196]
**Carrageenan**	Induction of apoptosis through upregulation of caspase-8, caspase-9 and caspase-3. Causes cell cycle arrest at G1, G2 or S phase.	[197,198]

## Abbreviations

GBM	Glioblastoma
TMZ	Temozolomide
MGMT	O^6^ methyl guanine DNA methyltransferase
MDM2	Mouse double minute 2 homolog
ARF	Alternative reading frame
PTEN	Phosphatase and tensin homolog
OXPHOS	Oxidative phosphorylation
IDH	Isocitrate dehydrogenase
CDK	Cyclin-dependent kinases
MAPK	Mitogen activated kinases
NF-κB	Nuclear factor kappa B
SGLT-1	Sodium-glucose transport protein 1
OATPs	Organic anion-transporting polypeptides
MMP	Matrix metalloproteinase
EGCG	Epigallocatechin gallate
PLGA	Poly (d, l-lactic-co-glycolic acid)
PEG	Polyethylene glycol
APE1	Apyrimidinic (AP) endonuclease 1
GSLCs	Glioma stem like cells
PDGFR	Platelet-derived growth factor receptor
EGFR	Epidermal growth factor receptor
IGF-1R	Insulin-like growth factor type 1 receptor
TIMP1	TIMP metallopeptidase inhibitor 1
